# Metformin repurposing in gout: enhancing febuxostat efficacy through anti-inflammatory and metabolic modulation: a randomized controlled double-blind study

**DOI:** 10.1007/s10787-026-02255-w

**Published:** 2026-05-30

**Authors:** Dalia N. Abdel-Sadek, Tarek M. Abd-Elazez, Sahar M. El-Haggar

**Affiliations:** 1 Clinical Pharmacy Department, Faculty of Pharmacy, Tanta University, Tanta, Egypt; 2 Department of Rheumatology and Rehabilitation, Faculty of Medicine, Al-Azhar University, New Damietta, Damietta, Egypt

**Keywords:** Gout, Febuxostat, Metformin, VAS, TNF-α, Uric acid

## Abstract

**Background:**

Gout is a chronic inflammatory disorder characterized by recurrent painful flares and elevated uric acid levels. Metformin possesses anti-inflammatory and metabolic regulatory properties that may offer additional benefit when combined with febuxostat.

**Aim:**

To evaluate the therapeutic efficacy of adding metformin to febuxostat in patients with gout.

**Methods:**

A randomized, double-blinded, controlled clinical trial was conducted on 60 gout patients and were randomly assigned to receive either febuxostat and placebo (control group, n = 30) or febuxostat plus metformin (metformin group, n = 30). Clinical assessment included the visual analogue scale (VAS) for pain, while laboratory evaluation comprised serum uric acid, tumor necrosis factor alpha (TNF-α), interleukin (IL)-1β, and free fatty acids. All biomarkers except free fatty acids were measured using ELISA; free fatty acids were determined colorimetrically. Lipid profile parameters were also recorded. Adverse effects were monitored in both groups.

**Results:**

Both groups showed significant reduction in all measured markers. The addition of metformin resulted in significantly greater reductions in VAS scores (− 33.3%), TNF-α (− 45.3%), IL-1β (− 12.3%), and uric acid levels (− 12.5%) compared with control group (all *p* < 0.05). The metformin group also demonstrated a significant decrease in free fatty acids (− 6.2%) with *p* = 0.032 and triglycerides (− 10.6%) with *p* = 0.002. No significant differences were observed regarding the frequency of drug-related side effects (all *p* > 0.05).

**Conclusion:**

Metformin as an adjuvant to febuxostat provides superior improvement in pain, inflammatory markers, uric acid levels, and metabolic parameters in gout patients, without increasing adverse effects.

## Introduction

Gout is a common inflammatory arthritis characterized by recurrent episodes of severe joint pain and swelling, resulting from the deposition of monosodium urate (MSU) crystals in synovial tissues (Ahn and So 2025). Although hyperuricemia is the biochemical hallmark of gout, the clinical manifestation of the disease is driven not solely by elevated urate levels but by a complex interplay between crystal deposition and the host’s inflammatory response. The global prevalence of gout continues to rise, partly due to the increasing burden of obesity, metabolic syndrome, and lifestyle-related risk factors (Herdiana et al. 2025).

The pathophysiology of gout involves two major processes: sustained hyperuricemia leading to MSU crystal formation, and crystal-induced activation of the innate immune system (Chen et al. 2025). MSU crystals trigger the NLRP3 inflammasome in monocytes and macrophages, resulting in the release of potent proinflammatory cytokines, particularly interleukin-1β (IL-1β) and tumor necrosis factor-α (TNF-α) (Tan et al. 2024). These cytokines promote neutrophil recruitment, amplify joint inflammation, and drive the intense pain characteristic of acute gout flares (Leask et al. 2024). Increasing evidence also suggests that persistent low-grade inflammation continues between attacks and contributes to comorbidities such as cardiovascular disease and insulin resistance (Ea et al. 2024).

Febuxostat, a xanthine oxidase inhibitor, is widely used to lower serum uric acid levels and prevent flare recurrence (Lee et al. 2025). However, some patients continue to experience residual inflammation, metabolic disturbances, and suboptimal symptom control despite adequate urate lowering (Yang et al. 2025). This highlights the need for adjunctive therapies that not only target uric acid metabolism but also modulate inflammatory and metabolic pathways.

Metformin, a widely used antihyperglycemic agent, has demonstrated pleiotropic effects beyond glucose control (Rehman et al. 2025). Mechanistically, metformin activates AMP-activated protein kinase (AMPK), which suppresses NF-κB signaling, reduces oxidative stress, and inhibits the production of proinflammatory cytokines such as TNF-α and IL-1β (Reed et al. 2025). It also improves lipid metabolism by lowering circulating free fatty acids and triglycerides and enhances endothelial function (Ding et al. 2021). Furthermore, metformin modulates immune cell activity, including the suppression of monocyte and macrophage inflammasome activation, and promotes autophagy, which may reduce crystal-induced inflammatory responses (Lin et al. 2023). Collectively, these actions suggest that metformin may mitigate both metabolic and inflammatory drivers of gout, potentially enhancing the therapeutic effect of urate-lowering therapy.

Given the central role of inflammation in gout pathogenesis and the demonstrated anti-inflammatory and metabolic benefits of metformin, combining metformin with febuxostat may yield superior clinical outcomes compared with febuxostat alone. We hypothesized that metformin adjunct therapy would result in greater reductions in inflammatory cytokines, uric acid levels, free fatty acids, and pain severity, and would also offer improvements in metabolic parameters without increasing adverse effects. The present randomized, double-blinded clinical trial aimed to evaluate the therapeutic effect of adding metformin to standard febuxostat therapy in patients with gout, focusing on changes in pain perception, inflammatory biomarkers, uric acid levels, metabolic markers, lipid profile, and drug-related side effects.

## Patients and methods

### Study design

**Fig. 1:**
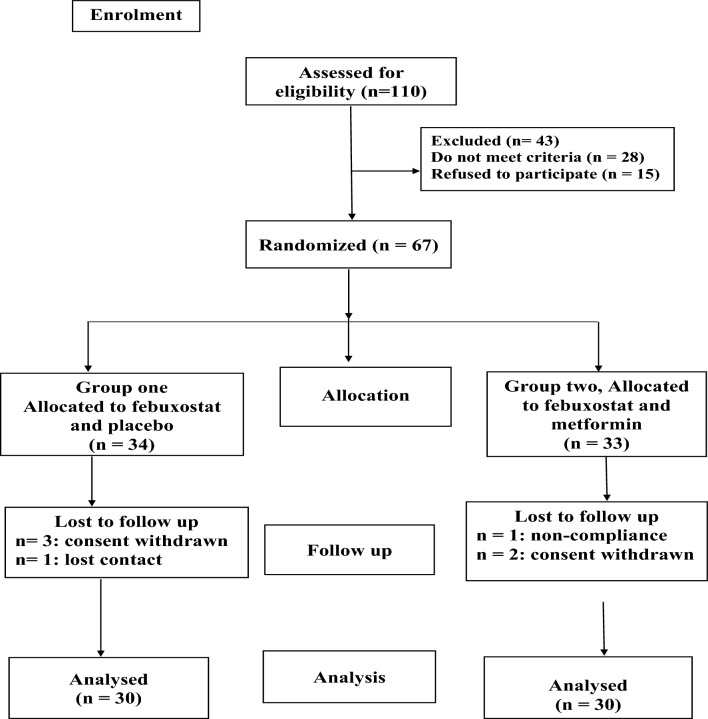
CONSORT diagram showing the flow of participants during the study

The trial was conducted between March 2025 and December 2025. Patients were recruited from the Rheumatology Department, Al-Azhar University Hospital, Damietta, Egypt. This was a randomized, double-blind, parallel-group clinical trial. Participants were allocated in a 1:1 ratio using a computer-generated block randomization sequence created by an independent statistician not involved in recruitment or assessment. Allocation concealment was ensured using sequentially numbered, opaque, sealed envelopes, which were opened only after participant enrollment (Fig. 1).

To maintain double blinding, both participants and investigators, including clinicians, outcome assessors, and laboratory personnel, were blinded to treatment allocation throughout the study. The study medications (metformin and placebo) were identical in appearance, packaging, and labeling and were dispensed by a pharmacist not involved in outcome assessment. Treatment codes were concealed until completion of data analysis.

### Inclusion criteria

Patients eligible for this study were adults aged 18 years or older of either sex, with a confirmed diagnosis of gout and serum uric acid levels above 7 mg/dL (Ulutaş et al. 2021). All participants were required to be obese, defined as having a body mass index (BMI) ≥ 30 kg/m^2^, to assess the combined effect of metabolic and inflammatory modulation in gout. Additionally, participants were required to have stable clinical status, without acute gout flares in the preceding two weeks, to ensure baseline comparability of inflammatory markers and pain assessment.

### Exclusion criteria

Patients with any form of diabetes mellitus were excluded to prevent confounding effects on metabolic and inflammatory markers. Individuals with drug-induced hyperuricemia—including those taking anti-tuberculosis agents (Mohapatra et al. 2021), low-dose aspirin (Ben Salem et al. 2017), cytotoxic chemotherapy, diuretics (XU and CUI 2020), immunosuppressants (Zheng et al. 2025), testosterone, or xylitol—were also excluded. Non-obese patients (BMI < 30 kg/m^2^) were ineligible, as the study focused on obesity-related metabolic dysregulation. Pregnant or lactating women were excluded for safety reasons. Additional exclusions included severe renal or hepatic impairment, active infections, uncontrolled cardiovascular disease, or any condition that could interfere with study participation or the interpretation of outcomes.

### Study outcomes

The primary outcomes of the study were changes in serum uric acid (sUA) levels and pain severity, assessed using the Visual Analogue Scale (VAS), to evaluate both biochemical and symptomatic improvement in gout patients.

Secondary outcomes included changes in inflammatory biomarkers, specifically tumor necrosis factor-α (TNF-α) and interleukin-1β (IL-1β), measured by ELISA, as well as metabolic parameters, including free fatty acids measured colorimetrically and lipid profile components (total cholesterol, triglycerides, LDL, and HDL).

### Sample size calculation

The sample size was calculated based on the primary outcomes of the study: change in serum uric acid (sUA) levels and pain severity assessed by the Visual Analogue Scale (VAS). Based on previous studies of febuxostat (Cutolo et al. 2017; Lin et al. 2019) in gout patients, a mean difference of 1.0 mg/dL in sUA and 1.5 points on the VAS, with standard deviations of 1.2 mg/dL and 1.8 points respectively, were considered clinically significant. Using a two-sided alpha of 0.05 and a power of 80%, the minimum required sample size was estimated to be 26 patients per group for each primary outcome. To account for potential dropouts and missing data, 30 patients were enrolled in each group, resulting in a total of 60 participants.

### Study intervention

Participants were randomly assigned to one of two treatment groups. Group 1 (Control group, n = 30) received febuxostat 80 mg (Feburic® 80 mg) once daily plus a matched placebo daily for six months. Group 2 (Metformin group, n = 30) received febuxostat 80 mg once daily in combination with metformin 1000 mg (Glucophage®) daily for six months. Both groups were instructed to maintain their usual diet and physical activity throughout the study period. Medication adherence was monitored through monthly follow-up visits and pill counts, and patients were advised to report any adverse effects immediately.

The selected dose of febuxostat (80 mg once daily) was chosen based on evidence from multiple clinical trials demonstrating its efficacy in lowering serum uric acid to target levels (< 6 mg/dL) in gout patients while maintaining a favorable safety profile (Becker et al. 2005; Xie et al. 2023). Phase II and III studies have shown that 80 mg daily provides an optimal balance between urate-lowering efficacy and risk of adverse events, with higher doses (e.g., 120 mg) reserved for patients not achieving target uric acid levels (Saag et al. 2019; Schumacher et al. 2008).

The metformin dose (1000 mg daily) was selected based on its established safety and tolerability in adults and its reported anti-inflammatory and metabolic effects in clinical studies (Binsaleh et al. 2025; El-Haggar et al. 2024). Previous studies in patients with metabolic syndrome (Orchard et al. 2005), obesity (Srinivasan et al. 2006), and rheumatoid arthritis (Gharib et al. 2021) have demonstrated that 1000 mg daily is sufficient to activate AMPK, reduce circulating free fatty acids, and attenuate proinflammatory cytokine production without significant gastrointestinal or systemic adverse effects. Lower doses may be subtherapeutic for metabolic modulation, while higher doses increase the risk of gastrointestinal intolerance.

### Study follow-up

Participants were closely monitored throughout the six-month study period to ensure adherence to the study protocol, maintain safety, and evaluate treatment efficacy. Follow-up included monthly in-person visits and periodic telephone check-ins to monitor symptoms, medication compliance, and adverse events. At baseline, all participants underwent a comprehensive medical evaluation, including liver and kidney function tests, to rule out contraindications to febuxostat or metformin.

Patients in both groups received standardized instructions regarding medication intake: febuxostat (80 mg) with or without metformin (1000 mg) daily, preferably at the same time each day, with a glass of water. They were advised not to alter doses, discontinue therapy, or start new medications without prior consultation with the study physician. Participants were counseled to maintain their usual diet and physical activity throughout the trial.

To ensure accurate assessment of therapeutic outcomes, patients were instructed to record daily symptoms in a study diary, including joint pain (VAS), gout flares, and any unusual symptoms or adverse events. Adverse events monitored included gastrointestinal disturbances, neurological symptoms (e.g., dizziness, headache), dermatologic reactions, and any other complaints potentially related to the study medications.

All participants were reminded of the importance of attending scheduled follow-up visits for clinical evaluation and laboratory testing, including serum uric acid, inflammatory biomarkers (TNF-α and IL-1β), free fatty acids, and lipid profile. Medication adherence was reinforced at each visit through verbal counseling and assessed by pill counts and patient self-reporting in diaries. Patients were also advised to avoid over-the-counter supplements targeting inflammation or uric acid during the study. Women of childbearing potential were instructed to use adequate contraception, and all participants were counseled to avoid excessive alcohol intake due to potential drug interactions and hepatotoxicity. All participants received standardized lifestyle and dietary counseling. This included advice tailored to the local cultural context, with emphasis on reducing the consumption of sugar-sweetened beverages and high-fructose foods, which are commonly consumed in hot climates and are associated with increased serum uric acid levels and inflammation. General recommendations regarding limiting alcohol intake were also provided in accordance with international gout management guidelines.

This structured follow-up ensured rigorous monitoring of both efficacy and safety outcomes, while promoting high adherence and reliable data collection throughout the study.

### Ethical considerations

The study was conducted in accordance with the Declaration of Helsinki and Good Clinical Practice guidelines. Ethical approval was obtained from the institutional review board of Al-Azhar University, Faculty of Medicine (Approval ID: 8/2022 PMRR2-3). All participants provided written informed consent prior to enrollment after receiving a detailed explanation of the study objectives, procedures, potential risks, and benefits. The trial was prospectively registered on ClinicalTrials.gov (Identifier: NCT06995339), ensuring transparency and adherence to international standards for clinical research. Participant confidentiality was maintained throughout the study, and data were handled in compliance with local regulations and institutional policies.

### Biochemical analysis

Blood samples were collected after an overnight fast, allowed to clot, and centrifuged at 3000 rpm for 10 min to obtain serum. All samples were processed immediately or stored at –20 °C until analysis.

Blood samples were collected from all participants at baseline and at the end of the study period for biochemical assessments. Serum levels of interleukin-1β (IL-1β) were measured using a PicoKine™ ELISA kit (Catalog Number: MBS175901, MyBioSource, USA), and tumor necrosis factor-α (TNF-α) was measured using an ELISA kit from Sunradio, Shanghai, China (Catalog Number: 201-12-0083), according to the manufacturers’ instructions.

Free fatty acids were quantified colorimetrically using a commercial kit (Catalogue Number: MAK466, Sigma-Aldrich, USA). All assays were performed in duplicate, and absorbance was measured using a microplate reader. Quality control samples and standard curves were included in each assay to ensure accuracy and reproducibility. Laboratory personnel were blinded to treatment allocation to minimize bias.

### Therapeutic assessment

Therapeutic efficacy was evaluated through both clinical and biochemical parameters. Clinical assessment focused on pain severity using the Visual Analogue Scale (VAS), a validated, self-reported 10—cm horizontal line where 0 indicates “no pain” and 10 represents “worst imaginable pain.” Participants were instructed to mark the point that best represented their average joint pain over the past 24 h. VAS scores were recorded at baseline and at the end of the study, and changes were used to assess improvement in gout-related pain (Langley and Sheppeard 1985).

Biochemical assessments included measurement of serum uric acid (sUA) to evaluate urate-lowering efficacy, inflammatory biomarkers (TNF-α and IL-1β) to assess anti-inflammatory effects, and free fatty acids, measured colorimetrically, to examine metabolic changes associated with therapy. Together, these clinical and biochemical measures provided a comprehensive evaluation of the symptomatic, inflammatory, and metabolic responses to febuxostat alone or in combination with metformin.

### Statistical analysis

Statistical analyses were conducted using GraphPad Prism version 9 (GraphPad Software, San Diego, CA, USA). The Shapiro–Wilk test was applied to evaluate the normality of continuous variables. For within-group comparisons, the paired Student’s *t*-test was employed for normally distributed data, whereas the Wilcoxon signed-rank test was used for non-parametric data. Between-group comparisons before and after treatment were performed using the unpaired Student’s t-test for parametric data and the Mann–Whitney U test for non-parametric data. Quantitative variables were presented as mean ± standard deviation (SD) when normally distributed, or as median with interquartile range (IQR) when non-parametric. Qualitative variables were summarized as frequencies and percentages, with categorical data analyzed using either the Chi-square test or Fisher’s exact test, as appropriate. Correlations were assessed using Pearson’s coefficient for normally distributed data and Spearman’s rank correlation for non-parametric data. To complement the statistical significance, we calculated the effect sizes (Cohen’s d) and 95% confidence intervals (CI) to assess the clinical impact of adding metformin to febuxostat therapy. All statistical tests were two-sided, with *p*-values < 0.05 considered statistically significant. Missing data in the study were minimal (< 5%) and were assumed to be missing at random (MAR). For analysis, a complete-case approach was employed, meaning that only participants with available data for each variable were included in the corresponding analysis.

## Results

### Clinical and demographic characteristics

The clinical and demographic characteristics of the two study groups are presented in Table 1. There were no statistically significant differences between the febuxostat group and the metformin group in any of the baseline parameters.

**Table 1:** Clinical and demographic data in the two study groups

parameter	Group (1) Febuxostat group (n = 30)	Group (2) Metformin group (n = 30)	*P*-value
Age (year)	43.63 ± 14.33	44.77 ± 11.86	0.739
Sex			0.602
M	16 (55.33%)	18 (60%)	
F	14 (46.67%)	12 (40%	
Height (m^2^)	1.787 ± 0.05	1.783 ± 0.06	0.803
Weight (kg)	109.4 ± 6.88	111.3 ± 5.92	0.255
BMI (kg/m^2^)	34.37 ± 3.13	35.09 ± 2.44	0.322
ALT (U/L)	28.60 ± 3.10	27.97 ± 3.04	0.428
AST (U/L)	30.13 ± 2.55	30.03 ± 3.61	0.902
SrCr (mg/dl)	1.009 ± 0.13	0.967 ± 0.15	0.257
Platelet count (10^3^)	170 ± 17.16	172.1 ± 12.04	0.579
Smoking (n)	4 (13.33%)	5 (16.66%)	0.525
FBG (mg/dl)	102.2 (92–105.4)	104 (98.70–108)	0.274
HA1C (%)	5.425 (5.17–5.52)	5.160 (4.87–5.52)	0.198
Disease duration (years)	2.22 (1.43–3.4)	1.87 (1.06–3.28)	0.589

Data are expressed as mean ± SD, numbers, percentages, median, and interquartile range, Control group, gout patients treated with febuxostat and placebo, Metformin group, gout patients treated with febuxostat plus metformin

*M* Male, *F* Female, *BMI* Body mass index, *ALT* Alanine amino-transferase, *AST* Aspartate amino-transferase, *SrCr*: Serum creatinine, *FBG* fasting blood glucose, *HA1C* Glycated haemoglobin

Significance at (*p* < 0.05)

The mean age of participants was comparable between the febuxostat group (43.63 ± 14.33 years) and the metformin group (44.77 ± 11.86 years; *p* = 0.739). Both groups showed a similar distribution of sex, with males representing 55.33% in the febuxostat group and 60% in the metformin group (*p* = 0.602).

Anthropometric measures, including height, weight, and BMI, did not differ significantly between groups. Mean BMI was 34.37 ± 3.13 kg/m^2^ in the febuxostat group and 35.09 ± 2.44 kg/m^2^ in the metformin group (*p* = 0.322).

Liver function markers (ALT and AST) and serum creatinine levels were comparable between the two groups, with no significant differences (*p* > 0.05). Platelet counts were also similar (170 ± 17.16 vs. 172.1 ± 12.04 × 10^3^; *p* = 0.579).

Regarding lifestyle factors, smoking prevalence did not differ significantly (13.33% vs. 16.66%; *p* = 0.525). Fasting blood glucose and HbA1c levels were comparable between groups, with no significant differences in FBG (*p* = 0.274) or HbA1c (*p* = 0.198). Overall, the two study groups were well matched at baseline, with no significant differences in demographic or clinical parameters. Disease duration was also comparable between the two study groups (p = 0.589).

### Effect of study medications on pain severity and biomarkers

Table 2 summarizes the effects of febuxostat alone and febuxostat plus metformin on pain scores and inflammatory and metabolic biomarkers.

**Table 2:** Effect of study medications on visual analogue scale and measured biomarkers

Character	Group (1) Febuxostat group (n = 30)			Group 2 Metformin group (n = 30)			*P* value
	Before treatment	After treatment	*P* value	Before treatment	After treatment	*P* value	After treatment
VAS	5 (4–6)	3 (1.75–4)	0.0001^*^	5 (3–5)	2 (1–2.25)	0.0001^*^	0.008^**^
TNF-α (pg/ml)	293 (253.3–316.3)	237.5 (127.8- 298.5)	0.039^*^	303 (272.5–325.5)	130 (104–249)	< 0.0001^*^	0.02**
IL-1β (pg/ml)	121.8 ± 28.17	91.35 ± 18.07	< 0.0001^#^	124.3 ± 22.61	80.13 ± 18.38	< 0.0001^#^	0.018^##^
Uric acid (mg/dl)	9.5 (8–10)	8 (7–9)	0.003^*^	10 (8–10)	7 (2.75–8)	0.0002^*^	0.008**
Free fatty acid (µmol/L)	242.6 ± 31.94	243.8 ± 33.58	0.630	255.5 ± 31.38	228.8 ± 23.49	0.006	0.032

Data was presented as numbers,mean and standard deviation, median and interquartile range, Control group, gout patients treated with febuxostat and placebo, Metformin group, gout patients treated with febuxostat plus metformin, VAS, visual analogue scale, TNF-α, tumor necrosis factor-alpha, IL-1β, interleukin-1 beta, (^*^) level of significance within the same group using Wilcoxon test. (^**^) level of significance between groups using Mann Whitney test. (^#^) level of significance within group using paired-t test. (^##^) level of significance between groups using unpaired-t test. Significance at (*p* < 0.05)

#### Pain assessment (VAS Score)

Both groups showed a significant reduction in pain severity after treatment. In the febuxostat group, VAS scores decreased from 5 (4–6) to 3 (1.75–4) (*p* = 0.0001). A more pronounced reduction was observed in the metformin group, where VAS scores declined from 5 (3–5) to 2 (1–2.25) (*p* = 0.0001). Importantly, the post-treatment VAS score was significantly lower in the metformin group compared with the febuxostat-only group (p = 0.008), indicating superior improvement in pain perception. A post hoc power analysis based on the observed effect sizes demonstrated that the study was adequately powered. For the primary outcome of pain severity (VAS), the estimated effect size (Cohen’s d ≈ 0.6) yielded a statistical power of approximately 80–85% with 30 participants per group.

#### Inflammatory markers (TNF-α and IL-1β)

TNF-α levels decreased significantly within both groups. Febuxostat alone reduced TNF-α from 293 (253.3–316.3) to 237.5 (127.8–298.5) pg/mL (*p* = 0.039), while the combination therapy produced a more substantial decline from 303 (272.5–325.5) to 130 (104–249) pg/mL (*p* < 0.0001). Post-treatment TNF-α levels were significantly lower in the metformin group than the febuxostat group (*p* = 0.02), indicating an enhanced anti-inflammatory effect.

Similarly, IL-1β levels showed significant reductions in both groups. Febuxostat alone decreased IL-1β from 121.8 ± 28.17 to 91.35 ± 18.07 pg/mL (*p* < 0.0001), whereas the metformin group showed a greater reduction from 124.3 ± 22.61 to 80.13 ± 18.38 pg/mL (*p* < 0.0001). Between-group comparison revealed significantly lower post-treatment IL-1β levels in the metformin group (*p* = 0.018).

#### Serum uric acid

Both treatments significantly improved uric acid levels. Uric acid decreased from 9.5 (8–10) to 8 (7–9) mg/dL in the febuxostat group (*p* = 0.003), while the combination group showed a larger reduction from 10 (8–10) to 7 (2.75–8) mg/dL (*p* = 0.0002). Post-treatment levels were significantly lower in the metformin group (*p* = 0.008), reflecting improved urate-lowering efficacy.

For serum uric acid, which showed a larger effect size (Cohen’s d ≈ 0.8–1.0), the corresponding power exceeded 90%.

#### Free fatty acids

In the febuxostat-only group, there was no significant change in free fatty acid levels after treatment (242.6 ± 31.94 vs. 243.8 ± 33.58 µmol/L; *p* = 0.630). In contrast, the metformin group demonstrated a significant decrease from 255.5 ± 31.38 to 228.8 ± 23.49 µmol/L (*p* = 0.006). Between-group analysis confirmed significantly lower post-treatment free fatty acid levels in the metformin group (*p* = 0.032).

### Clinical relevance and effect size analysis

The addition of metformin to febuxostat therapy resulted in moderate-to-large clinically meaningful effects on patient-reported pain (VAS: d = 0.87, 95% CI [0.34, 1.40]), inflammatory markers (TNF-α: d = 0.52, 95% CI [0.006, 1.03]; IL-1β: d = 0.62, 95% CI [0.10, 1.13]), and serum uric acid (d = 0.71, 95% CI [0.18, 1.24]). Free fatty acid levels also showed a moderate effect (d = 0.52, 95% CI [0.004, 1.03]). These results support that, beyond statistical significance, the reductions observed are clinically relevant, reinforcing the therapeutic potential of metformin as an adjunct in gout management.

### Effect of study medications on lipid profile

Table 3 summarizes the changes in lipid parameters before and after treatment in both groups. In the febuxostat group, no significant changes were observed in total cholesterol (TC), triglycerides (TG), LDL, or HDL levels (all *p* > 0.05). In contrast, the metformin group demonstrated a significant reduction in TC (*p* = 0.003) and TG (*p* < 0.0001), while changes in LDL and HDL were not statistically significant. Between-group comparisons revealed no significant differences in post-treatment TC, LDL, or HDL levels (all *p* > 0.05). However, post-treatment TG levels were significantly lower in the metformin group compared to the febuxostat group (*p* = 0.002), indicating a favorable effect of metformin on triglyceride levels.

**Table 3:** Comparison of lipid profile between the two groups

Character	Group (1) Febuxostat group (n = 30)			Group 2Metformin group (n = 30)			*P* value
	Before treatment	After treatment	*P* value	Before treatment	After treatment	*P* value	After treatment
TC (mg/dl)	162.1 ± 14.33	161.4 ± 14.38	0.246	165.4 ± 12.87	158.9 ± 13.64	0.003^*^	0.46
TG (mg/dl)	132.7 ± 12.26	132.1 ± 11.6	0.667	129.5 ± 11.44	118.6 ± 11.84	< 0.0001^*^	0.002**
LDL (mg/dl)	91.85 ± 15.54	93.61 ± 16.07	0.547	91.85 ± 15.54	88.71 ± 13.94	0.39	0.223
HDL (mg/dl)	43.67 ± 6.83	41.40 ± 7.24	0.356	44.5 ± 7.45	46.50 ± 7.75	0.247	0.719

Data was presented as numbers, median and interquartile range (mean and standard deviation), Control group, gout patients treated with febuxostat and placebo, Metformin group, gout patients treated with febuxostat plus metformin, (TC), total cholesterol; (TG), triglycerides; (HDL), high-density lipoprotein; (LDL), low-density lipoprotein. (^*^) level of significance within group using paired-t test. (^**^) level of significance between groups using unpaired-t test. Significance at (*p* < 0.05)

### Correlation analysis in the metformin group

Correlation analysis among the measured variables in the metformin-treated group is presented in Table 4. Significant positive correlations were observed between uric acid levels and all other studied parameters. Uric acid showed strong correlations with IL-1β (r = 0.663, *p* < 0.0001) and VAS (r = 0.793, *p* < 0.0001), indicating that higher uric acid levels were associated with greater inflammatory burden and higher pain scores. Moderate correlations were also found between uric acid and free fatty acids (r = 0.328, *p* = 0.011), as well as TNF-α (r = 0.321, *p* = 0.012).

**Table 4:** Correlation analysis between measured variables in metformin group

Variables	Uric acid		Free fatty acid		TNF-α		IL-1β		VAS	
	r	p	r	p	r	p	r	p	r	p
Uric acid		0.328	0.011	0.321	0.012	0.663	< 0.0001	0.793	< 0.0001	
Free fatty acid	0.328	0.011		0.271	0.036	0.340	0.007	0.408	0.001	
TNF-α	0.321	0.012	0.271	0.036		0.517	0.0001	0.388	0.002	
IL-1β	0.663	< 0.0001	0.340	0.007	0.517	0.0001		0.705	< 0.0001	
VAS	0.793	< 0.0001	0.408	0.001	0.388	0.002	0.705	< 0.0001		

(r) correlation coefficient. Significance at (*p* < 0.05)

*VAS* visual analogue scale, *TNF-α* tumor necrosis factor-alpha, *IL-1β* interleukin-1 beta

Free fatty acids demonstrated significant positive correlations with TNF-α (r = 0.271, *p* = 0.036), IL-1β (r = 0.340, *p* = 0.007), and VAS (r = 0.408, *p* = 0.001), suggesting an association between metabolic dysregulation, inflammation, and pain intensity.

TNF-α was positively correlated with IL-1β (r = 0.517, *p* = 0.0001) and VAS (r = 0.388, *p* = 0.002), confirming that higher levels of inflammatory cytokines were linked to worse clinical symptoms.

IL-1β also demonstrated a strong correlation with VAS (r = 0.705, *p* < 0.0001), indicating that increased interleukin-1β levels were markedly associated with higher pain severity.

Overall, these findings show a coherent pattern in which elevations in uric acid, inflammatory biomarkers, and free fatty acids are strongly associated with increased patient-reported pain scores in the metformin group.

### Multivariable linear regression analysis of predictors of post-treatment clinical and biochemical outcomes

Multivariable linear regression analysis was performed to evaluate the independent effect of metformin on changes in clinical and biochemical outcomes after adjusting for age, sex, gout duration, and baseline values (Table 5). The addition of metformin was identified as a significant independent predictor of improvement in multiple outcomes, including ΔVAS (β = 0.910, *t* = 2.507, *p* = 0.015), ΔIL-1β (β = 14.033, *t* = 3.353, *p* = 0.001), Δ serum uric acid (β = 1.344, *t* = 3.325, *p* = 0.002), and Δ free fatty acids (β = 20.360, *t* = 3.201, *p* = 0.002). A borderline association was observed for ΔTNF-α (β = 61.807, *t *= 1.991, *p* = 0.051). In contrast, age, sex, and gout duration were not significant predictors across all models (all *p* > 0.05). Baseline values were consistently strong predictors of their respective changes, particularly for VAS, TNF-α, IL-1β, uric acid, and FFA (all *p* ≤ 0.001). These findings demonstrate that the beneficial effects of metformin are independent of demographic and disease-related factors, supporting its role as an effective adjunct therapy in gout management.

**Table 5:** Multivariable linear regression analysis of predictors of post-treatment clinical and biochemical outcomes

Outcome	Predictor	β (Coefficient)	*t*-value	*p*-value
ΔVAS	Group (Metformin)	0.910	2.507	0.0152*
	Age	0.0078	0.565	0.5741
	Sex	0.335	0.920	0.3619
	Gout duration	0.064	0.390	0.6978
	VAS baseline	0.927	5.910	< 0.001*
ΔTNF-α	Group (Metformin)	61.807	1.991	0.0515
	Age	0.951	-0.790	0.4332
	Sex	− 48.395	-1.519	0.1345
	Gout duration	15.196	1.063	0.2923
	TNF baseline	0.980	5.493	< 0.001*
ΔIL-1β	Group (Metformin)	14.033	3.353	0.00147*
	Age	0.072	0.450	0.6547
	Sex	− 3.606	− 0.845	0.4018
	Gout duration	− 2.812	− 1.483	0.1439
	IL-1 baseline	0.740	9.217	< 0.001*
Δ Uric Acid	Group (Metformin)	1.344	3.325	0.00159*
	Age	− 0.014	− 1.067	0.2904
	Sex	− 0.314	− 0.761	0.4501
	Gout duration	0.153	0.793	0.4314
	Uric acid baseline	0.545	3.469	0.00103*
ΔFFA	Group (Metformin)	20.360	3.201	0.00229*
	Age	0.012	0.048	0.9620
	Sex	9.773	1.534	0.1309
	Gout duration	− 4.573	− 1.593	0.1170
	FFA baseline	0.486	4.873	< 0.001*

Statistically significant at *p* < 0.05. Δ indicates change from baseline to post-treatment

*VAS* visual analogue scale, *TNF-α* tumor necrosis factor-alpha, *IL-1β*, interleukin-1 beta, *FFA* free fatty acid

### Drug-related side effects

The comparison of adverse effects between the two study groups revealed no statistically significant differences for any reported side effect (all *p* > 0.05). Vomiting occurred in 5 patients in the metformin group and 4 patients in the control group (*p* = 0.717). Nausea was reported in 4 patients receiving metformin and 2 patients in the control group (*p* = 0.389).

Diarrhea was slightly more frequent in the metformin group (6 patients) compared to the control group (3 patients), but this difference was not significant (*p* = 0.278). Skin rash occurred in 2 patients on metformin versus 4 patients on febuxostat alone (*p* = 0.389). Headache was reported in 4 patients in the metformin group and 5 in the control group (*p* = 0.717).

Flatulence affected 5 patients in the metformin group and 3 patients in the control group (*p* = 0.447), while dizziness occurred in 5 and 4 patients, respectively (*p* = 0.717). (Table 6).

**Table 6:** Analysis of drug related side effects between the studied groups

Side effect	Control group (n = 30)	Metformin group (n = 30)	*P* value
Vomiting	4	5	0.717
Nausea	2	4	0.389
Diarrhoea	3	6	0.278
Skin rash	4	2	0.389
Headache	5	4	0.717
Flatulence	3	5	0.447
Dizziness	4	5	0.717

Data was presented as numbers. Control group, gout patients treated with febuxostat and placebo, Metformin group, gout patients treated with febuxostat plus metformin. Significance at (*p* < 0.05) using Chi square or Fisher exact test as appropriate

## Discussion

Gout is a chronic inflammatory disorder characterized by hyperuricemia and deposition of monosodium urate crystals in joints, leading to pain, swelling, and progressive joint damage. Traditional therapies focus primarily on urate-lowering agents, such as xanthine oxidase inhibitors, and anti-inflammatory drugs for acute flares. However, the persistent inflammatory and metabolic derangements in gout, especially in obese patients, highlight the need for novel therapeutic strategies that target both urate metabolism and systemic inflammation (Timsans et al. 2024).

Drug repurposing, the strategy of identifying new therapeutic uses for existing medications, has emerged as a promising approach in modern clinical research (Alarfaj et al. 2023**; **Alarfaj et al. 2025**; **AlRasheed et al. 2025a**; **AlRasheed et al. 2025b; Omar et al. 2025). This strategy offers several advantages, including established safety profiles, known pharmacokinetics, and reduced development time and cost compared with de novo drug discovery. Numerous drugs originally developed for specific indications have been successfully repurposed across diverse diseases; for example, metformin, initially approved for type 2 diabetes, has been explored in cancer (Brown et al. 2020), cardiovascular disease (Griffin et al. 2017), polycystic ovary syndrome (Bridger et al. 2006), and autoimmune disorders. Similarly, colchicine, originally used for gout flares, has shown benefit in cardiovascular disease (Robinson et al. 2022), and statins, developed for hyperlipidemia, have demonstrated anti-inflammatory effects in rheumatoid arthritis (Li et al. 2018) and other inflammatory conditions (AlRasheed et al. 2025b).

In this study, treatment with febuxostat alone significantly reduced sUA levels and improved pain scores (VAS) in obese gout patients, confirming its well-established urate-lowering and symptomatic efficacy. These results are consistent with previous clinical trials, where febuxostat 80 mg/day achieved target sUA (< 6 mg/dL) in 60–80% of patients over 6–12 weeks and significantly reduced gout flares and pain scores compared with placebo or allopurinol (Perez-Ruiz et al. 2016). For example, the Phase III trials by Becker et al. and Schumacher et al. demonstrated robust reductions in sUA and improved patient-reported outcomes with febuxostat therapy (Becker et al. 2010; Schumacher et al. 2008).

The combination of febuxostat with metformin produced a greater reduction in both sUA and VAS scores, suggesting a synergistic effect of metformin in modulating both metabolic and inflammatory pathways. To our knowledge, retrospective studies have investigated the effect of metformin in gout (Marrugo et al. 2024; Veenstra et al. 2022); however, the findings align with the broader concept of drug repurposing and the metabolic modulation by metformin. The improvement in VAS scores observed in both groups reflects the clinical efficacy of febuxostat in controlling gout flares, likely through reduction of urate crystal formation and subsequent joint inflammation. These results were in line with previous studies that investigated the effect of metformin on pain scores in various diseases (El-Haggar et al. 2024; Pan et al. 2025).

Serum uric acid was significantly reduced in metformin group compared to control group. These observation were in line with previous reports (Veenstra et al. 2022; Younis et al. 2021). Febuxostat is a selective xanthine oxidase inhibitor, directly reducing urate production (Guma et al. 2022). The additional reduction in sUA observed with the combination therapy could be related to metformin’s metabolic effects, including improved insulin sensitivity and modulation of renal urate excretion (Veenstra et al. 2022). Several previous clinical and observational studies have explored the relationship between metformin use and serum uric acid levels, with generally supportive but somewhat heterogeneous findings. Some studies have reported that metformin therapy is associated with modest reductions in serum uric acid levels, particularly in patients with type 2 diabetes and insulin resistance (Barskova et al. 2009, 2005). Beyond its well-established anti-inflammatory and metabolic effects, metformin has been reported to influence serum uric acid levels through several complementary mechanisms. First, metformin improves insulin sensitivity and reduces hyperinsulinemia, which is known to impair renal urate excretion by downregulating urate transporters in the proximal renal tubules (Wang and Wang 2025). Second, metformin-mediated activation of AMP-activated protein kinase (AMPK) may modulate hepatic purine metabolism, indirectly reducing urate production (Goel et al. 2022; Jin et al. 2022). By enhancing insulin signaling, metformin facilitates renal uric acid clearance, thereby lowering circulating urate levels. Third, by lowering circulating free fatty acids (FFAs), metformin attenuates FFA-induced inflammatory stress in the kidneys, further promoting urate excretion and reducing renal inflammation (Li et al. 2024). Renal urate reabsorption is primarily mediated by URAT1 and GLUT9 transporters in the proximal tubules, which play a major role in determining serum uric acid levels. Genetic and functional studies confirm that alterations in SLC22A12 and SLC2A9 significantly influence urate handling and hyperuricemia risk (Ali 2026). Metabolic dysfunction such as insulin resistance enhances renal urate reabsorption via increased activity of URAT1 and GLUT9, contributing to elevated serum uric acid levels (Deji-Oloruntoba et al. 2025). Metformin improves insulin sensitivity, which has been associated with reductions in serum uric acid in clinical studies, suggesting an indirect mechanism through which improved metabolic control may decrease urate reabsorption and enhance uricosuria (Wang and Wang 2025). Collectively, these mechanisms suggest that metformin not only acts systemically to reduce metabolic and inflammatory drivers of gout but also directly contributes to urate lowering, complementing the xanthine oxidase inhibition of febuxostat. This dual action may explain the greater reductions in sUA observed in patients receiving combination therapy.

Taken together, these findings suggest that metformin exerts a dual effect in obese gout patients: directly improving metabolic parameters that indirectly affect uric acid handling, and modulating inflammatory pathways that contribute to pain and joint inflammation. This may explain the superior clinical outcomes in the combination group compared with febuxostat alone.

In addition to clinical improvement and urate reduction, our study demonstrated significant decreases in TNF-α and IL-1β levels in both groups, with a more pronounced effect in the metformin group. TNF-α and IL-1β are central mediators of gout-related inflammation, driving leukocyte recruitment, synovial inflammation, and joint pain (Zhang et al. 2021). Elevated levels of these cytokines correlate with disease severity and symptom intensity (Peral-Garrido et al. 2025; Zeng et al. 2022), which aligns with our finding of a strong positive correlation between VAS scores and cytokine levels in the metformin group.

Previous studies have shown that febuxostat monotherapy can reduce inflammatory cytokines indirectly by lowering uric acid and thereby decreasing monosodium urate crystal formation. For instance, it was reported that reduced IL-1β and TNF-α in hyperuricemic patients treated with febuxostat (Huang et al. 2020; Kraev et al. 2023). Mechanistically, metformin activates AMPK, a key energy-sensing kinase that inhibits NF-κB signaling, leading to decreased transcription of pro-inflammatory cytokines, including TNF-α and IL-1β (Hattori et al. 2006; Tsuji et al. 2020). In parallel, metformin suppresses NLRP3 inflammasome activation in monocytes and macrophages, directly reducing IL-1β release, which is central to gout pathogenesis (Cao et al. 2025; Hosseini et al. 2025). Metformin also improves insulin sensitivity, which enhances renal urate excretion and complements the urate-lowering effect of febuxostat (Veenstra et al. 2022). By lowering circulating free fatty acids and triglycerides, metformin reduces metabolic-driven inflammation (Castro Cabezas et al. 2012), indirectly attenuating cytokine production and oxidative stress.

Similarly, free fatty acids (FFAs), which are elevated in obesity and metabolic syndrome, were significantly reduced in the metformin group but not in the febuxostat-alone group. Elevated FFAs are known to activate pro-inflammatory pathways, including NF-κB and NLRP3 inflammasome activation, contributing to systemic inflammation and potentially enhancing urate crystal-induced joint inflammation (Legrand-Poels et al. 2014; Ma et al. 2023; Zhang et al. 2019). By reducing FFAs, metformin may indirectly attenuate cytokine production, complementing its direct anti-inflammatory effects (Castro Cabezas et al. 2012; Patanè et al. 2000). This mechanistic link explains the observed correlations between FFAs, cytokines, and VAS scores in our study, highlighting the interplay between metabolic and inflammatory pathways in obese gout patients.

Collectively, these findings support a dual mechanism of action for the combination therapy: febuxostat lowers uric acid and reduces crystal-induced inflammation, while metformin targets systemic metabolic dysregulation and directly inhibits inflammatory signaling. This synergy may explain the superior reductions in VAS, sUA, and inflammatory biomarkers compared with febuxostat alone.

Although lipid parameters were within the normal range at baseline, the observed reductions—particularly in triglycerides and free fatty acids—may reflect early metabolic improvements rather than correction of overt dyslipidemia. In this context, these changes are better interpreted as markers of improved insulin sensitivity and metabolic regulation, which are particularly relevant in obese patients with gout who are at increased cardiometabolic risk. Importantly, the reduction in free fatty acids is more plausibly explained by decreased adipose tissue lipolysis and enhanced insulin action, rather than being secondary to triglyceride lowering (Mason et al. 2025). This aligns with the known metabolic effects of metformin, including suppression of hepatic gluconeogenesis and modulation of lipid metabolism via AMP-activated protein kinase activation (Khodadadi et al. 2022). Therefore, the lipid-modifying effects observed in this study may have implications for cardiovascular risk reduction, even in patients with baseline lipid values within the normal range.

These results highlight the metabolic benefits of repurposing metformin in obese gout patients, where systemic lipid dysregulation exacerbates inflammation and disease severity. By improving TG and FFA levels, metformin may indirectly attenuate cytokine-mediated inflammation and synergize with febuxostat to optimize clinical outcomes.

Correlation analysis in the metformin group revealed significant positive relationships between VAS scores, serum uric acid, free fatty acids (FFAs), and pro-inflammatory cytokines (TNF-α and IL-1β). The strongest correlation was observed between VAS and IL-1β, highlighting the central role of IL-1β in mediating gout-related pain. Similarly, the strong correlation between VAS and sUA, supports the well-established link between hyperuricemia and symptom severity (Scirè et al. 2016).

The correlations between FFAs and both inflammatory markers and VAS scores suggest a metabolic–inflammatory interplay in obese gout patients. Elevated FFAs can activate the NLRP3 inflammasome and NF-κB pathway, promoting TNF-α and IL-1β release, which in turn exacerbates joint inflammation and pain (Wani et al. 2021). By reducing FFAs, metformin may disrupt this cycle, attenuating cytokine production and clinical symptoms.The observed correlations also support the hypothesis that metabolic dysregulation amplifies inflammatory responses in gout. Improvements in metabolic markers (FFAs, TGs) were associated with reductions in cytokine levels and pain, providing mechanistic evidence for the synergistic effects of combining febuxostat with metformin. These findings are consistent with previous studies in obesity-related inflammatory disorders, where modulation of metabolic parameters led to decreases in systemic inflammation and symptomatic improvement (Ma et al. 2023; Wani et al. 2021).

The multivariable regression analysis confirms the robustness and independence of the observed treatment effects. After adjustment for age, sex, gout duration, and baseline values, metformin remained a significant independent predictor of improvement in VAS, IL-1β, serum uric acid, and free fatty acids, with a borderline effect on TNF-α. These findings indicate that the benefits of metformin are not driven by baseline differences or patient characteristics, but rather reflect a true therapeutic effect.

Notably, age, sex, and gout duration were not significant predictors across models, suggesting a consistent treatment response across patient subgroups. Baseline values were strong predictors of change, which is expected and supports the validity of the models. Mechanistically, these results are consistent with the known metabolic and anti-inflammatory effects of metformin, particularly its role in improving insulin sensitivity and modulating inflammatory pathways (Cameron et al. 2016; Kristófi and Eriksson 2021). Collectively, these findings reinforce the potential of metformin as an effective adjunct therapy targeting both metabolic and inflammatory aspects of gout.

Both treatment regimens were generally well tolerated. The most frequently reported adverse events in the metformin plus febuxostat group included gastrointestinal symptoms such as diarrhea, nausea, vomiting, and flatulence, as well as headache and dizziness. Importantly, the incidence of these events did not differ significantly from the febuxostat-alone group (all *p* > 0.05), indicating that the addition of metformin at 1000 mg daily did not increase the overall risk of adverse effects. No severe or life-threatening events were reported, and all adverse events were transient and resolved without discontinuation of therapy. Our results were in line with previous studies (Binsaleh et al. 2025; El-Haggar et al. 2024).

These findings are consistent with the established safety profile of metformin, which is generally well tolerated in non-diabetic populations at standard doses, with gastrointestinal discomfort being the most common limitation (El-Haggar et al. 2024). Similarly, febuxostat has been shown to be safe in previous randomized trials, with mild adverse effects such as headache and liver enzyme elevations reported in a small proportion of patients (Kimura et al. 2018; Suzuki et al. 2021). The combination therapy did not exacerbate these known risks, suggesting that febuxostat and metformin can be co-administered safely in obese gout patients.

Overall, the safety data support the feasibility of using metformin as an adjunct to febuxostat for both metabolic modulation and anti-inflammatory benefits, without compromising patient tolerability. These findings reinforce the potential clinical utility of drug repurposing strategies in gout, particularly for patients with comorbid obesity and metabolic dysregulation.

This study has several notable strengths. First, it is a randomized, double-blind clinical trial, minimizing selection and observer bias and enhancing the reliability of the findings. Second, we included comprehensive clinical and biochemical assessments, including VAS, serum uric acid, inflammatory cytokines (TNF-α, IL-1β), and metabolic markers (free fatty acids, triglycerides), which allowed us to explore both symptomatic and mechanistic effects of therapy. Third, the study focused on obese gout patients, a population in which metabolic dysregulation exacerbates inflammation and urate retention, making the investigation of repurposed metabolic therapy particularly relevant. Finally, the combination of febuxostat with metformin represents a novel therapeutic strategy, highlighting the translational potential of drug repurposing for inflammatory-metabolic disorders.

Despite these strengths, several limitations should be acknowledged. The sample size was relatively small (n = 60), which may limit the generalizability of the findings and reduce the statistical power to detect rare adverse events or smaller effect sizes. The follow-up period of six months was sufficient to observe short-term clinical and biochemical changes; however, it may not fully capture the long-term sustainability of urate reduction, metabolic improvements, or the frequency of recurrent gout flares. Therefore, future large-scale, multicenter studies with extended follow-up durations are warranted to confirm the persistence of these benefits and to assess their impact on disease progression and comorbidities.

Another limitation of this study is the lack of systematic blood pressure assessment. As blood pressure is a core component of metabolic syndrome, its absence limits the comprehensive evaluation of cardiometabolic risk among participants. Future studies should incorporate standardized blood pressure measurements to allow a more complete characterization of metabolic syndrome and its interaction with inflammatory and metabolic biomarkers.

This study was conducted at a single center, which may limit external validity. Additionally, although correlations suggest mechanistic links between metabolic markers and inflammation, causality cannot be definitively established. The mechanistic discussion remains limited and should be explored in future research. Finally, certain methodological details, including the specifics of randomization, allocation concealment, and handling of missing data, were not fully reported, which should be addressed to enhance reproducibility and study transparency.

## Conclusion

In summary, this study demonstrates that febuxostat effectively reduces serum uric acid and alleviates pain in obese gout patients, and that the addition of metformin enhances these effects through complementary anti-inflammatory and metabolic mechanisms. Combination therapy significantly lowered pro-inflammatory cytokines (TNF-α, IL-1β), free fatty acids, and triglycerides, correlating with improved clinical outcomes. The interventions were well tolerated, with no significant increase in adverse events.

These findings highlight the potential of drug repurposing strategies, such as metformin, to target both metabolic dysregulation and inflammation in gout, offering a novel, mechanistically informed approach to improve patient outcomes, particularly in obese populations. Further large-scale, multicenter studies with longer follow-up are warranted to confirm these benefits and explore the long-term impact on gout progression and comorbid metabolic disorders.

## Data Availability

Data is available upon request.
